# Comparative analysis of machine learning approaches to classify tumor mutation burden in lung adenocarcinoma using histopathology images

**DOI:** 10.1038/s41598-021-95747-4

**Published:** 2021-08-16

**Authors:** Apaar Sadhwani, Huang-Wei Chang, Ali Behrooz, Trissia Brown, Isabelle Auvigne-Flament, Hardik Patel, Robert Findlater, Vanessa Velez, Fraser Tan, Kamilla Tekiela, Ellery Wulczyn, Eunhee S. Yi, Craig H. Mermel, Debra Hanks, Po-Hsuan Cameron Chen, Kimary Kulig, Cory Batenchuk, David F. Steiner, Peter Cimermancic

**Affiliations:** 1 Google Health, Palo Alto, CA USA; 2 Verily Life Sciences, South San Francisco, CA USA; 3 Google Health via Vituity, Emeryville, CA USA; 4 Department of Laboratory Medicine and Pathology, Mayo Clinic, Rochester, MN USA; 5 Present Address: PathPresenter Corp., New York, NY USA

**Keywords:** Machine learning, Non-small-cell lung cancer, Tumour biomarkers, Cancer imaging

## Abstract

Both histologic subtypes and tumor mutation burden (TMB) represent important biomarkers in lung cancer, with implications for patient prognosis and treatment decisions. Typically, TMB is evaluated by comprehensive genomic profiling but this requires use of finite tissue specimens and costly, time-consuming laboratory processes. Histologic subtype classification represents an established component of lung adenocarcinoma histopathology, but can be challenging and is associated with substantial inter-pathologist variability. Here we developed a deep learning system to both classify histologic patterns in lung adenocarcinoma and predict TMB status using de-identified Hematoxylin and Eosin (H&E) stained whole slide images. We first trained a convolutional neural network to map histologic features across whole slide images of lung cancer resection specimens. On evaluation using an external data source, this model achieved patch-level area under the receiver operating characteristic curve (AUC) of 0.78–0.98 across nine histologic features. We then integrated the output of this model with clinico-demographic data to develop an interpretable model for TMB classification. The resulting end-to-end system was evaluated on 172 held out cases from TCGA, achieving an AUC of 0.71 (95% CI 0.63–0.80). The benefit of using histologic features in predicting TMB is highlighted by the significant improvement this approach offers over using the clinical features alone (AUC of 0.63 [95% CI 0.53–0.72], p = 0.002). Furthermore, we found that our histologic subtype-based approach achieved performance similar to that of a weakly supervised approach (AUC of 0.72 [95% CI 0.64–0.80]). Together these results underscore that incorporating histologic patterns in biomarker prediction for lung cancer provides informative signals, and that interpretable approaches utilizing these patterns perform comparably with less interpretable, weakly supervised approaches.

## Introduction

Tumor Mutation Burden (TMB) has emerged as an important biomarker for predicting response to immunotherapy in lung cancer as well as other solid tumors^1–3^. TMB is defined as the relative number of somatic mutations present in a tumor, with high TMB associated with favorable immunotherapy response, likely due to increased expression of neo-antigens and corresponding immunogenicity of the tumor microenvironment^4^. Additionally, while TMB has emerged as a valuable component of precision medicine, it requires genomic profiling of the tumor which is costly, time consuming, and requires sufficient tissue, leading to limited availability for many patients. Additionally, due to differences in coverage regions, bioinformatics pipelines, and strategies for counting mutations, substantial variability can still exist in regards to the TMB count across laboratories^5^.

Several recent efforts have explored the potential for deep learning algorithms to perform a range of tasks in pathology, including the detection of tumor, the classification of tumor histology, and more recently, the prediction of molecular biomarker status^6–9^. These approaches show promise, but the histopathological features being learned and used by the algorithms are often unknown and cannot be quality-controlled or readily verified by researchers and pathologists. Building on previously described association of TMB with both histologic subtype^10,11^ and smoking status^2^, we hypothesized that a computational approach aimed at predicting TMB status from H&E images could benefit both in transparency and performance by utilizing known, clinically relevant features.

For lung adenocarcinoma, histologic subtype represents an established framework with diagnostic and prognostic significance^12,13^. Current guidelines call for the reporting of lepidic, acinar, papillary, micropapillary and solid subtypes in 5% increments^12^ with emerging clinical significance for cribriform subtype as well^14^. However, high inter-rater variability in the assessment of these categories can limit overall utility^15,16^. As such, automated subtype classification thus has the potential to provide more accurate and consistent classification, and initial efforts demonstrate promising results^17^. We reasoned that a computational approach enabling comprehensive, detailed classification of histologic features across all tissue regions may provide insights into underlying biological processes, including those associated with high TMB.

In this study, we first developed and evaluated a convolutional neural network (CNN) for classification of lung adenocarcinoma histologic subtypes across all regions of digitized whole slide images (WSIs). We then developed a second model to predict the TMB status using the output from the histologic subtype CNN combined with additional clinical data. The result is a deep learning system capable of predicting TMB status while also providing an interpretable and informative feature map of the tumor histology. In parallel, we developed an end-to-end, “weakly supervised” deep learning model for TMB classification, which uses only the case-level TMB information and the slide image to train the model, and thus provides less insights into the features used to predict TMB. Importantly, this allowed development of strategies to combine models and comparison across several possible modeling approaches.

## Methods

### Modeling approaches

**Figure 1:**
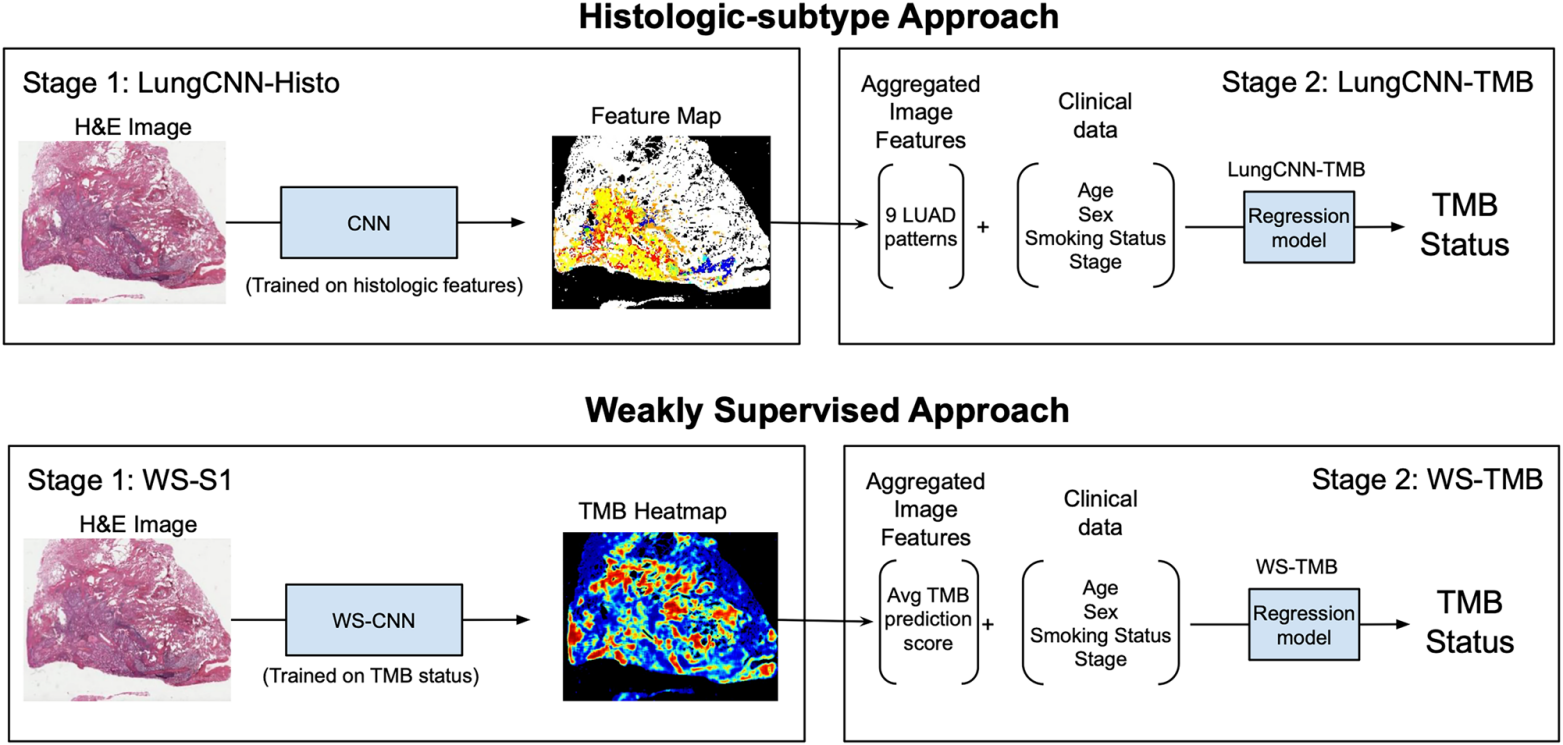
Overview of modeling approaches and inference. Overview of the histologic-subtype approach (top) and the weakly supervised approach (bottom). In the histologic-subtype approach, feature maps of histologic patterns produced by LungCNN-Histo in stage 1 and are combined with clinical features to predict TMB-status in stage 2. The weakly supervised approach also utilizes a two stage approach, whereby a weakly supervised model (WS-S1) is developed to produce patch-level TMB predictions at inference. The patch-level data are aggregated over the entire tissue area in stage 1 and combined with clinical features to provide TMB classification in stage 2.

The two general modeling approaches are summarized in Fig. 1. In the first approach, we explored the use of known histological features by designing a two-stage system. For the first stage (LungCNN-Histo), we developed a CNN to classify known histologic features for individual patches across hematoxylin and eosin (H&E) stained whole slide images (WSIs). In the second stage (LungCNN-TMB), the patch-level CNN predictions from LungCNN-Histo are aggregated over the entire slide and combined with clinical features (smoking status, age, stage, sex) to classify the TMB status. Binary classification of TMB status was chosen to reflect the clinical application of TMB in predicting treatment response^1^ (see also TMB Status section below).

In contrast to the histologic subtype approach utilizing known lung adenocarcinoma features, we also developed a weakly supervised approach by using the case-level TMB count as the only available label during model training. To allow comparisons and hybrid models, we adopted a two-stage approach for the weakly supervised model as well. In the first stage, a weakly supervised model (WS-S1) was developed to directly infer TMB status for individual patches using case level labels. Patch-level predictions were aggregated across the WSI to produce a “TMB prediction score”. In the second stage (WS-TMB), the TMB prediction score was combined with clinical features to classify the TMB status. Additional details can be found in the “model development” section below.

### Data

This study utilized digitized, de-identified WSIs from two independent datasets. The first comprised all lung adenocarcinoma (LUAD) images from The Cancer Genome Atlas (TCGA). All TCGA LUAD images and associated clinical and mutation count data are managed by the NCI and NHGRI and were accessed via the Genomic Data Commons Portal (https://gdc.cancer.gov) along with the associated TCGA publications^18,19^. The second dataset, consisting of 50 de-identified H&E WSIs from 50 lung adenocarcinoma cases, was obtained from an independent pathology laboratory in the United States (hereafter referred to as Dataset 2 or DS2). Most images from TCGA were scanned at a resolution of 0.25 μm/pixel (40×) with a subset scanned at 0.50 μm/pixel (20×). DS2 images used in this study were scanned at 40× (0.5 μm/pixel). All research was performed in accordance with relevant guidelines and regulations. The study was approved by the institutional review board of Advarra (Columbia, Maryland) and deemed exempt from informed consent as all data and images were deidentified. This IRB covered the use of de-identified cases independent of data source for the purposes of this study.

The training and testing data used for all models in this work are summarized in Table 1 and Supplemental Figure S1. Available slides were partitioned by case into train and test sets for the LungCNN-Histo and TMB classification models, respectively. The resulting data splits are referred to as Histo-Train, Histo-Test, TMB-Train, WS-Train, and TMB-Test and are summarized in Table 1. While some of these sets contain overlapping cases, Histo-Train and Histo-Test are non-overlapping and TMB-Test represents a completely held out set of cases, non-overlapping with any other data splits. LungCNN-Histo was trained on annotated slides from TCGA (Histo-Train) and validated on annotated slides from both TCGA and DS2 (Histo-Test). LungCNN-TMB, the second stage model for TMB classification, and WS-TMB, the second stage model of the weakly supervised approach, were both trained using the same TCGA data (TMB-Train), which required availability of TMB and clinical features. The first stage of the weakly supervised TMB model was trained using WS-Train, which comprises all available TCGA cases with TMB counts available that were not assigned to TMB-Test. All TMB classification models were evaluated using the same set of held out TCGA cases as one another (TMB-Test). Lastly, a subset of TMB-Test represents held out cases that are also from held out TCGA tissue source sites (referred to as “unseen sites” since no cases from these sites were used for model development).

**Table 1:** Summary of data used for training and testing the LungCNN-Histo and the TMB classification models.

Model		LungCNN-Histo			WS-S1	LungCNN-TMB and WS-TMB		
Dataset name		Histo-Train	Histo-Test		WS-Train	TMB-Train	TMB-Test	
Data source		TCGA LUAD	TCGA LUAD	DS2	TCGA LUAD	TCGA LUAD	TCGA LUAD (unseen sites)	TCGA LUAD (seen sites)
Number of cases (number of slides)		64 (68)	38 (40)	50 (50)	317 (261)	242 (295)	84 (84)	88 (93)
Number of tissue source sites		22	20	N/A	23	21	10	17
Age range (median)		38–84 (67.5)	48–83 (68.5)	40–70 (56)	33–87 (67)	33–87 (67)	42–84 (64)	41–88 (69)
Pathologic stage	I	37	20	35	148	138	40	56
	II	15	11	9	60	54	27	17
	III	8	6	6	37	35	10	12
	IV	4	1	0	15	15	7	3
	N/A	0	0	0	1	0	0	0
Sex	Female	40	17	17	144	134	44	49
	Male	24	21	33	117	108	40	39
Smoking status	Non-smoker	26	17	0	96	89	38	36
	Smoker	30	21	0	157	153	46	52
	N/A	8	0	50	8	0	0	0
TMB status	Low	47	28	N/A	182	167	61	59
	High	17	10	N/A	79	75	23	29

### Annotations

Annotations of specific regions corresponding to 9 LUAD histologic features were performed manually. These include 6 tumor subtypes (acinar, lepidic, solid, papillary, micropapillary, cribriform), 2 microenvironment features (necrosis, leukocyte aggregates), and “other”, which represents primarily non-tumor features such as normal lung tissue and tumor stroma.

Annotations for model training were provided by three individuals, first a trained research scientist (molecular biology PhD training with 5 years of experience in lung histopathology research), followed by review by a US board-certified pathologist (> 20 years experience) and additional confirmation by a thoracic pathology subspecialist (> 20 years experience). Annotations were non-exhaustive for each WSI, with the goal of capturing many examples and patches for all classes even though all tumor on each slide was not annotated. These annotations were performed by identifying regions of the image corresponding to each feature of interest. Internally developed image annotation software was used, allowing downstream use of the annotated patches. All of the patches annotated in this manner were used for training, with at most one annotation per patch. This process involved approximately 60 min per slide for the research scientist and approximately 20 min per slide for the reviewing pathologists. The quantity of training annotations and patches for each class are summarized in Supplemental Table S1.

To enable rigorous and quantitative patch-level evaluation, annotations for model evaluation were provided by a cohort of 7 US board certified pathologists (non-thoracic, median years of post-training experience 12, range 3–28), with 3 pathologists independently annotating each slide. For this process, regions of interest (ROI; 1 mm × 1 mm) were first pre-selected by one pathologist per slide (4–7 ROIs per slide) with guidance to capture a diverse set of morphological features. These ROIs were then independently annotated by 3 pathologists to provide labels for the nine histologic features specified above (resulting in at most one annotation per patch per pathologist). The ROIs were labeled using internally developed labeling software to allow “drag and drop” selection of the specified 1 mm × 1 mm bounding box.

### TMB status

To reflect the clinical application of TMB in predicting response to immunotherapy, we focused on the binary classification of TMB status as TMB-high versus TMB-low^1^. In this study, a prespecified threshold of 323 mutations was used throughout for both model development and model evaluation, utilizing mutation counts provided for TCGA cases^18,19^. This threshold corresponds to the 70th percentile of the TCGA LUAD cohort, representing a value consistent with the portion of cases expected to be above the clinically validated threshold of 10 mutations/megabase^1^. The percentile-based strategy was used because mapping of TMB using whole exome sequencing to that obtained using a companion diagnostic tumor profiling panel is complicated by many factors, including mutation types counted, call filters, and coverage regions^5^. However, depending on the mutation calling and counting strategies, roughly 200 mutations on whole exome sequencing has also been proposed as an approximation of the clinically relevant threshold^20^. As such, we also performed secondary analysis using a threshold of 200 for TMB-high vs. TMB-low.

### Model development

#### Histologic-subtype approach—lung histology model (LungCNN-Histo)

In the first stage of the end-to-end deep learning system, we developed a model to classify lung histologic patterns. Specifically, we segmented WSIs into small non-overlapping patches of 8 × 8 μm at 10 × magnification and trained a CNN to classify each patch into the 9 annotated classes, including 6 tumor subtypes and 2 microenvironment features. We used the Inception V3 architecture^21^ for the CNN model, training on regions of 512 × 512 μm (512 × 512 pixels) to provide additional context for classifying the central 8 × 8 μm patch.

This model is trained on annotations corresponding to 4.6 million patches across 68 TCGA slides from Histo-Train. We used softmax cross-entropy as the loss function and identified the optimal model hyperparameters using cross-validation (Supplemental Table S2). A magnification of 10× (approximately 1 μm/pixel) was selected based on consistent performance across all classes. Regularization (via dropout, weight decay and color perturbations) was found to be beneficial. The final model was obtained by ensembling 10 independently trained replicas trained on the entire set of annotations, and calibrated on the remaining 8 slides in Histo-Train by reweighting the class scores. The model outputs class scores over the 9 classes, the highest of which is interpreted as the model predicted class. At inference, this produces a heatmap of LungCNN-Histo predictions for the entire WSI, which is used to validate the model, visualize its outputs, and as input for the downstream second stage TMB model.

#### Histologic-subtype approach—tumor mutation burden model (LungCNN-TMB)

The second stage of the end-to-end system is LungCNN-TMB, which was trained using the TMB-Train set (295 slides across 242 TCGA cases). The TMB model aggregates the morphological features captured from the first stage over the WSI and combines them with clinical features to estimate the TMB status for each case using logistic regression. Specifically, it first quantitates the fraction of the nine classes aggregated over the WSI as predicted by LungCNN-Histo. Next, we normalized the quantitation of the 6 tumor subtypes to represent subtype pattern composition as a fraction of the total predicted tumor content. From the clinical data, we input smoking status, pathologic stage, age, and sex as provided by TCGA. These 13 features—9 image features and 4 clinical features—are normalized and input to a logistic regression model that predicts the TMB status. The image features and age (in years) are treated as continuous variables, pathologic stage (I–IV) as ordinal, and smoking status and sex as categorical. We used softmax cross-entropy as the loss function and identified the set of features and optimal hyperparameters for the model using cross-validation (Supplemental Table S3). For comparison, we additionally fit a logistic regression model using only the 4 clinical features.

#### Weakly supervised approach—stage 1 model (WS-S1)

We also developed a weakly supervised model using the case-level TMB status as training labels. This model predicts the TMB status directly from the WSI, using a training pipeline described previously^22^. The model consists of multiple convolutional neural networks (CNNs) that share weights and an average pooling layer, averaging the outputs from each CNN before feeding into a fully-connected layer. The choice of an average pooling layer in this design enables flexibility in utilizing a varying number of input patches to make a prediction. At training, we sample a fixed number of patches from the tissue area for the slide uniformly randomly and run them through the model to produce a “TMB prediction score” for the slide. This model was trained using the WS-Train set (317 slides across 261 TCGA cases; Table 1) using a patch size of 512 × 512 at 10 × magnification. Preliminary experiments evaluated a range of resolutions up to 40× (0.25 μm/pixel), but as these results were inferior to 10 × and the complete dataset was not available with 40 × scans, the final hyperparameter search did not include 40×. Hyperparameter details are available in Supplemental Table S4. We used the MobileNet^23^ architecture for the CNN, which is lightweight and adapts well to the limited data available for training this weakly supervised model. We employed significant regularization and early stopping to avoid overfitting.

#### Weakly supervised approach—tumor mutation burden model (WS-TMB)

In the second stage of the weakly supervised approach (WS-TMB), the TMB prediction score is combined with the four clinical features (smoking status, pathologic stage, age, and sex) in a logistic regression model that predicts the TMB status. This parallels the development of LungCNN-TMB, and uses the same train and test sets to enable comparison and integration across approaches (Fig. 1). Using this framework, we additionally developed a hybrid approach that combines the TMB prediction score, the 9 histology features (from LungCNN-Histo) and the four clinical features in a logistic regression model.

### Model evaluation

#### Histologic-subtype approach—lung histology model (LungCNN-Histo)

The trained model was evaluated on the held out set of 40 TCGA slides (TCGA-Histo-Test) and an external validation set of 50 slides (DS2). Each slide of the test set was annotated independently by three pathologists (see “Annotations” section above for details). To ensure high-quality reference standard data for model evaluation across canonical examples of the selected features, we formed a consensus patch set (3.1 million patches of size 512 × 512 μm) containing only those patches with agreement across the three pathologists. Patch-level model performance was assessed for each histology feature by measuring the area under the receiver operating characteristic curve (AUC) on this consensus patch set. We additionally evaluated error modes of the model via a confusion matrix of the classifications over this set.

Complementing this, we performed a concordance analysis to better understand the inter-pathologist agreement for interpretation of the nine histologic patterns, using Fleiss’ kappa. In contrast to the consensus patch set, this analysis considered all patches annotated by any three pathologists. To further evaluate inter-pathologist agreement, we also performed exploratory concordance subanalyses focused on two groups of features—the tumor group containing the six pathologic tumor subtypes, and the non-tumor group containing the three additional features. We computed kappa for both the tumor and non-tumor groups by performing analysis over the patches that received only tumor labels or non-tumor labels, respectively. Lastly, percent of patches with consensus for each feature was also calculated. All patches with annotations from three pathologists were included for this analysis and percent consensus was calculated as the number of patches with a consensus label divided by the total number patches receiving at least one label for the same feature.

#### Histologic-subtype approach—LungCNN-TMB

LungCNN-TMB is evaluated on TMB-Test (n = 177 slides from held out TCGA cases not previously used for model development). Planned subanalysis was also performed on cases from the unseen sites within TMB-Test (n = 84 slides). The primary analysis was calculating AUC for prediction of TMB-high versus TMB-low cases with additional analysis to compare performance to that of using the clinical features alone.

#### Weakly supervised approach—WS-TMB

WS-TMB is evaluated on TMB-Test, which aids comparison with LungCNN-TMB. The primary analysis involves assessing the AUC for predicting the TMB status and comparing this with the AUC for LungCNN-TMB. Variants of this approach using different sets of features, such as the hybrid approach, all employ the same test set for consistency.

### Statistical analysis

We use the Scikit-learn Python library for all analysis in this work^24^. We used fivefold cross-validation to determine the optimal hyperparameters for the tumor histology model (LungCNN-Histo). For the final trained model, the one-vs-rest AUC for all classes were computed at the patch level. The AUC confidence intervals were estimated using (non-parametric) bootstrapping by drawing 1000 samples, obtained by resampling the slides in the dataset with replacement. The slide-level resampling ensures independent sampling of clusters, while preserving the correlation among patches within each slide.

We evaluated the inter-pathologist concordance over the nine histologic patterns in LungCNN-Histo using Fleiss’ kappa. Concordance was assessed using Fleiss’ kappa as it allows for different subsets of (3) pathologists to label each patch in a non fully-crossed design. The confidence intervals for kappa were estimated via bootstrapping by drawing 1000 samples as before.

For predicting the TMB status using any combination of the input features—image features from the tumor histology model, prediction scores from the weakly supervised model, and clinical features—we identified and trained (multivariate) logistic regression models using the same method for each set of features. We first identified the optimal set of features and hyperparameters via tenfold cross validation. Next, the chosen logistic regression model was trained, its coefficients in this multivariate setting were obtained, and the AUC on the test set was computed. Finally, statistical inference was performed. All p-values are one-sided, unless noted otherwise. Whenever bootstrapping was employed, we used 1000 independent samples obtained by resampling the cases. The p-value of the AUC was determined using a permutation test and confidence intervals were estimated via bootstrapping. The (two-sided) p-values for the model coefficients were estimated using (parametric) bootstrapping. The confidence intervals for the model coefficients were estimated by refitting the model each time on the bootstrapped samples. We employed a paired permutation test to test for the improvement in AUC offered by the model combining LungCNN-Histo and clinical features over the model using clinical features only.

## Results

### Histologic subtype and feature classification

**Figure 2:**
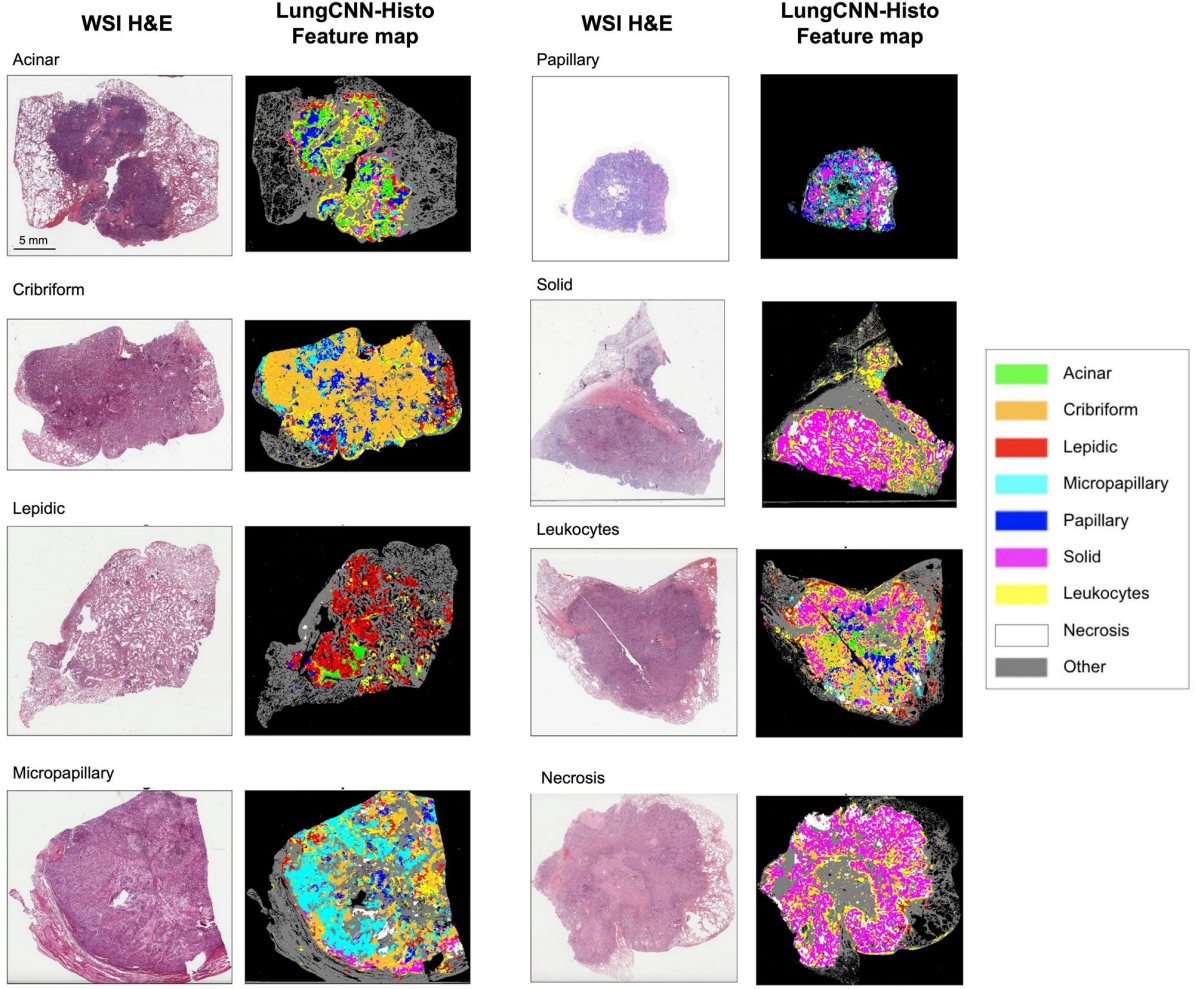
Visualization of LungCNN-Histo predictions for whole slide images. Shown are representative whole slide images with examples of each of the feature classes. *WSI* whole slide image, *H&E* hematoxylin and eosin.

**Figure 3:**
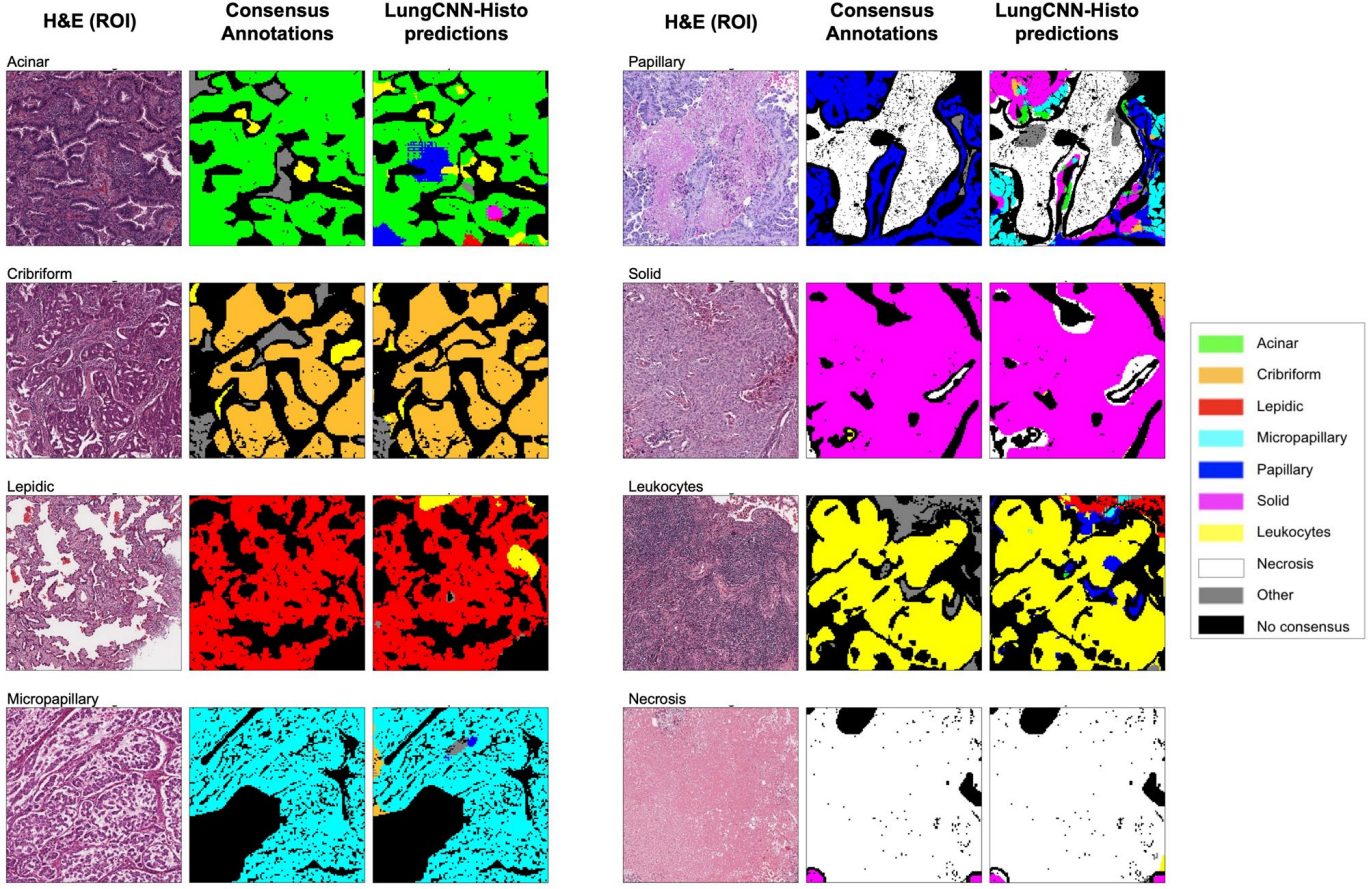
Visualization of LungCNN-Histo predictions for annotated regions. Shown are representative regions of interest (1 mm × 1 mm) for regions with predominance of each of the feature classes (see legend). Black regions represent areas of tissue where there was lack of consensus for the three pathologist annotations. Middle columns for each panel represent consensus pathologist annotations and the right panel depicts the model classification across patches (with the non-consensus patches masked in black to match for easier visual reference to the pathologist annotations.) *H&E* hematoxylin and eosin, *ROIs* regions of interest.

The histologic pattern model (LungCNN-Histo) was evaluated across the 9 classes for which it was trained (acinar, lepidic, solid, papillary, micropapillary, cribriform, necrosis, leukocyte aggregates, and “other”). As described in the “Methods” section, LungCNN-Histo was evaluated using held out cases from TCGA (TCGA-Histo-Test) as well as an external validation set (DS2). The area under the receiver operating characteristic curve (AUC) for each class across both data sources is summarized in Table 2. For TCGA, the average AUC across features was 0.93 (range: 0.85 to 0.99). For the external validation set, DS2, the average AUC across features was 0.92 (range: 0.67 to 0.99). Among tumor subtypes specifically, for both TCGA and DS2 slides, highest performance was observed for micropapillary and solid subtypes and the lowest performance for papillary subtypes. We note, however, that the number of pappillary patches in the consensus DS2 set was relatively small (Supplemental Table S1), resulting in wide confidence intervals for this class. For TCGA the AUC for leukocyte aggregates was 0.96 (95% CI 0.95–0.98) and for necrosis was 0.98 (95% CI 0.97–0.99). For DS2 the AUC for leukocyte aggregates was 0.99 (95% CI 0.98–0.99) and for necrosis was 0.98 (95% CI 0.98–0.99). Example visualizations of the LungCNN-Histo predictions across WSIs as well as annotated regions of interest are shown in Figs. 2 and  3, respectively. Generally highest performance was observed for non-tumor patterns with error modes that included misclassification of acinar regions as papillary, misclassification of cribriform as solid, and misclassification of papillary as lepidic or micropapillary (Supplemental Figure S2).

**Table 2:** LungCNN-Histo performance.

Histologic feature	AUC (95% CI)		
	Combined	TCGA LUAD	DS2
Acinar	0.87 (0.84, 0.90)	0.88 (0.83, 0.92)	0.88 (0.83, 0.92)
Lepidic	0.95 (0.95, 0.99)	0.97 (0.96, 0.99)	0.94 (0.92, 0.96)
Solid	0.96 (0.95, 0.97)	0.97 (0.95, 0.98)	0.95 (0.92, 0.97)
Papillary	0.78 (0.64, 0.85)	0.85 (0.72, 0.91)	0.67 (0.53, 0.96)
Micropapillary	0.97 (0.95, 0.99)	0.99 (0.98, 0.99)	0.96 (0.91, 0.98)
Cribriform	0.93 (0.89, 0.97)	0.92 (0.86, 0.97)	0.94 (0.87, 0.99)
Necrosis	0.98 (0.97, 0.99)	0.98 (0.97, 0.99)	0.99 (0.98, 0.99)
Leukocyte aggregates	0.98 (0.97, 0.99)	0.96 (0.95, 0.98)	0.99 (0.98, 0.99)
Other	0.90 (0.89, 0.92)	0.89 (0.86, 0.91)	0.92 (0.90, 0.94)

The area under the receiver operating characteristic curve (AUC) for the 9 patterns predicted by LungCNN-Histo at the patch-level.

The inter-pathologist concordance for the annotated patches was assessed using Fleiss’ kappa. The patch-level kappa for the nine-way classification of the histologic patterns was 0.64 (95% CI 0.60–0.68). For the task of classifying patches as tumor or not, the kappa was 0.96 (95% CI 0.95–0.97). The kappa for the six histologic subtypes was 0.37 (95% CI 0.30–0.42), underscoring the variability among pathologists for labeling these tumor subtypes. The kappa for the 3 non-tumor categories was 0.88 (95% CI 0.83–0.92). Consistent with these results, the overall percent consensus was lower for the tumor patches than for the other features (Supplemental Figure S3).

### Tumor mutation burden prediction

**Figure 4:**
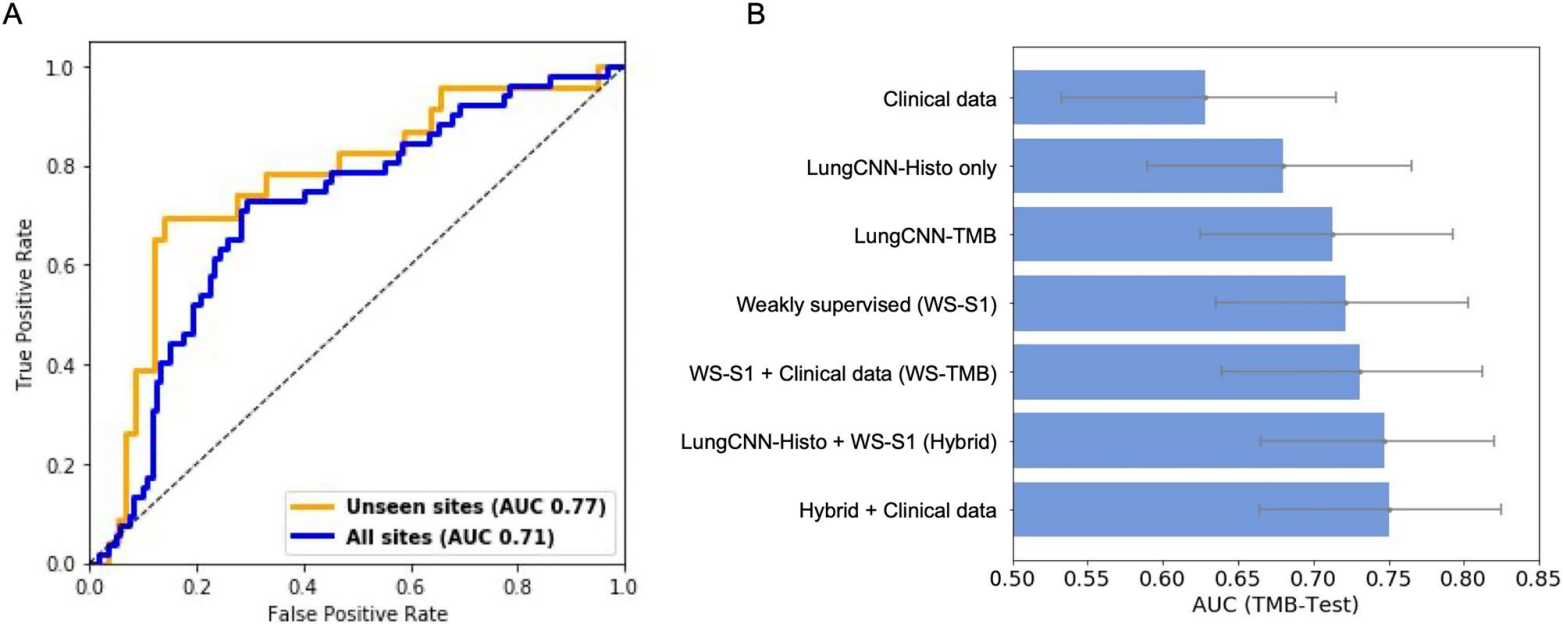
Model performance for classification of TMB. **(A)** Receiver operator characteristic curves (ROCs) and corresponding AUCs for LungCNN-TMB in predicting TMB status across all sites of the test set (blue) and the subset of cases from unseen sites (orange). **(B)** AUCs are plotted for TMB classification across all models developed. Clinical data plus LungCNN-Histo represents LungCNN-TMB. Error bars are 95% confidence intervals.

Performance of the LungCNN-TMB model, which combines clinical data and histologic pattern predictions, is summarized in Fig. 4A. On TMB-Test (the set of all held out TCGA cases), LungCNN-TMB achieved an AUC of 0.71 [95% CI 0.63–0.79] for TMB-high vs. TMB-low classification. Compared to use of clinical features alone (AUC of 0.63 [95% CI 0.53–0.72]), incorporation of the histologic features from LungCNN-Histo provided significant improvement (p = 0.002). On the cases from the unseen TCGA sites, LungCNN-TMB achieved an AUC of 0.77 [95% CI 0.64–0.88] for TMB classification. The clinical features alone demonstrated an AUC of 0.66 [95% CI 0.51–0.79]. At an operating point chosen with equal weight on sensitivity and specificity, the LungCNN-TMB performance corresponds to a sensitivity of 78.3% and a specificity of 77.6% for the cases from unseen sites and a sensitivity of 71.2% and specificity of 71.7% for the complete test set.

We also trained a weakly supervised model to directly predict TMB from WSIs. We evaluated the aggregated output of this model (WS-S1) as a stand-alone feature as well as combined with the clinical features (WS-TMB). The corresponding AUCs for the different approaches are summarized in Fig. 4B. The WS-TMB model (weakly supervised TMB prediction score combined with clinical features) demonstrated an AUC of 0.72 [95% CI 0.64–0.80]. We also developed a “hybrid model” approach to combine outputs of the weakly supervised TMB model with those of the strongly supervised LungCNN-Histo model as well as the 4 clinical features. This approach achieved an AUC of 0.75 (95% CI 0.66–0.83). Overall, these results demonstrate a trend towards increased performance with the combination of clinical factors, histologic pattern quantitation, and features extracted via weakly supervised learning, suggesting independent predictive value for all of these features.

The overall performance of all models was also evaluated for the alternative TMB threshold of 200 mutations, near the median value for the TCGA LUAD cohort. The same performance trends were observed across the different models at this threshold, although overall performance was generally lower, potentially representing the more challenging task of discriminating between cases with intermediate TMB (Supplemental Figure S4).

To better understand the features of LungCNN-TMB contributing to the TMB prediction, we evaluated the coefficients of the second stage logistic regression model and corresponding odds ratio (see “Methods” for details). In this analysis the absolute value of coefficients represents the level of association with TMB-high classification. Amongst the features of the histologic-subtype approach (LungCNN-TMB), solid and papillary subtypes were found to be most informative for TMB prediction, with solid subtype as the most independently informative (coefficient = 1.13; Table 3). The AUCs for independent features, including stage 1 of the weakly supervised approach (WS-S1), were also calculated and are summarized in supplemental Figure S5A, with the highest independent predictive value associated with the weakly supervised model output and the solid subtype feature. Also, while multiple histologic features were associated with predicted TMB status, in the presence of solid subtype the quantity of other subtypes was less informative, due largely to the correlation of subtypes with one another. The correlation of histologic subtypes is summarized in supplemental Figure S5B, showing both strong positive and negative correlation for several feature pairs. When considering clinical features alone, smoking status, pathologic stage, and age were all found to be independently informative for predicting TMB status. In the LungCNN-TMB model (which combines histologic features and clinical features), of the clinical features, smoking status had the strongest association with TMB (Coefficient 0.90, p = 0.002; Table 3).

**Table 3:** Coefficients and odds ratios for individual features of the LungCNN-TMB model.

	Feature	Estimate (95% CI)	p-value	OR
Model coefficients	Solid	1.13 (0.49, 1.73)	0.001*	3.10
	Smoking status	0.90 (0.41, 1.36)	0.002*	2.46
	Age	−0.86 (−1.44, −0.29)	0.062	0.42
	Pathologic stage	−0.51 (−1.12, 0.09)	0.157	0.60
	Intercept	−1.56 (−1.90, −1.22)	0.000*	–

Two-sided P-values were estimated using bootstrapping. A logistic regression model combines features from the 9 image features of LungCNN-Histo and 4 clinical features (age in years, sex, pathologic stage, smoking status) to predict TMB status. The image features (quantified as proportion of total tumor area) and age (in years) are treated as continuous variables, pathologic stage (I–IV) as ordinal, and smoking status (binary) and sex (M,F) as categorical. Features in this table represent those providing best model via cross-validation (see “Methods”).

*OR* odds ratio

Finally, as dataset size may have implications for modeling approach selection, we performed a data titration experiment to provide insights regarding the impact of the dataset size on the weakly supervised approach in particular, summarized in supplemental Figure S6. We observed an expected decrease in performance when less training data was utilized. However, there appeared to be decreasing marginal performance gains at our final training dataset size, suggesting the performance may not increase substantially for the weakly supervised approach, even with more data.

## Discussion

Estimating TMB in lung cancer has important implications for immunotherapy treatment options. Current strategies for evaluating TMB require comprehensive genomic profiling which can be costly and slow, with turn around times often on the order of weeks, potentially delaying treatment decisions. In this study, we explored the ability to rapidly estimate TMB status in lung adenocarcinoma using H&E histopathology images combined with clinico-demographic data. The resulting end to end system provides both histologic subtype classification and interpretable TMB status estimation with an AUC of 0.77 on a set of slides from held out sites, significantly better than using the baseline clinical features alone (AUC of 0.66 for the same cases) and on par with TMB prediction models recently reported for other cancer types from TCGA^9,25^.

Advances and contributions of this work include the following. First, this work involves the development and rigorous evaluation of a patch-level model for clinically relevant histologic features in lung adenocarcinoma. Second, our results demonstrate that interpretable prediction of molecular biomarkers based on established histologic patterns is feasible and comparable to more difficult to explain deep learning approaches. Additionally, we provide extensive comparison of modeling approaches for this task, including development and evaluation of a weakly supervised model, the integration of clinical features, and hybrid models combining weakly supervised feature extraction with information from established histologic subtype predictions. Given the important role of model explainability in both establishing user trust and facilitating regulatory processes^26^, understanding performance tradeoffs of different approaches has meaningful implications for potential use of AI-based biomarker predictions in clinical practice.

Initial efforts have shown early promise for molecular prediction from H&E images using weakly supervised deep learning approaches (see review from Echle et al.^7^). The present study provides a direct comparison of a weakly supervised approach with a novel, histologic subtype-based approach for TMB classification**.** Our results demonstrate that for datasets of this size (which represents a common range of data availability for data of this type), the two stage approach may provide a superior option overall, providing advantages of direct interpretability and biological insights with little tradeoff in terms of performance. Specific possible use cases for the interpretable end-to-end system described here include use as a surrogate for TMB if genomic profiling is not available, to prioritize initial treatment selection while awaiting molecular results, or to triage cases for molecular profiling if tissue or testing resources are limited.

Additionally, because it utilizes clinically relevant histologic features, with further clinical evaluation LungCNN-Histo itself has the potential to be a useful tool for classification of lung adenocarcinoma subtype in a manner that is more quantitative and standardized than current approaches. Additionally, it provides an opportunity for extension to additional “second stage” prediction targets in future work such as other molecular biomarkers, patient prognosis, or treatment response. We performed patch level validation of LungCNN-Histo in order to rigorously evaluate performance across canonical examples of selected features and demonstrate sufficient accuracy for downstream use in the second stage TMB prediction model. However, patch level subtype labeling itself is not a clinical task and proved challenging, with high inter-pathologist variability. Further work to define ground truth definitions of tumor subtypes at the patch level may both facilitate model improvements and enable applications such as automated or assisted tumor subtyping, case triage, quality assurance, or research into clinical and biological significance of spatial relationships across tumor features. Weakly supervised learning on the other hand has the advantages of not requiring expert feature annotations, and thus may be better suited for application to large datasets with readily available case-level labels, especially for use cases when interpretability may be less critical. Looking forward, utilizing interpretable approaches to investigate or validate regions of interest identified by weakly supervised models, or potentially using models trained on interpretable features as pre-training to initialize weakly supervised models remain interesting areas to explore. Weakly supervised approaches that operate on both the raw image pixels as well as the output from models trained on known features also represent a potential future direction. Lastly, especially for use cases in which there is human interaction with model predictions in histopathology, identifying and communicating potential causal or biological associations between machine learned features and model predictions may become increasingly useful^27^. In this case, such efforts may involve techniques to map molecular findings to specific regions highlighted by the models in order to demonstrate underlying biology associated with learned features and predictions.

We also observed that hybrid models utilizing weakly supervised learning combined with models trained on known histologic patterns may provide superior performance. Notably, this indicates that the weakly supervised model is learning at least some features independent from the known histologic patterns. Further, the improved performance of the AI-based approaches relative to the clinical features alone demonstrate that both deep learning approaches have learned histologic features independent of clinical variables themselves. Taken together, this work provides a novel proof of concept for the field to further explore optimal use cases for such hybrid models, whereby weakly and strongly supervised deep learning approaches may complement one another.

To develop a system with the most practical relevance for current pathology workflows and interpretability, we focused our study on images of slides from formalin fixed paraffin embedded (FFPE) tissue. This has the benefit of providing TMB and histologic subtype predictions that can be readily evaluated by pathologists and applied to routine histopathology slides. While there does not appear to be a generalizable performance advantage for FFPE versus frozen section images for deep learning based molecular predictions^28^, many other efforts have focused on frozen sections for TMB prediction^7^. Future work comparing and contrasting the performance and relevant features for TMB prediction using FFPE versus frozen specimens may enable these different approaches to inform and complement one another.

Limitations of this study include the following. While we evaluated LungCNN-Histo on an external validation set (DS2) and our TMB test set included unseen TCGA tissue source sites, further validation on additional datasets remains warranted. In particular, while TCGA data can provide useful evaluation of generalization across the different sites, the specimens themselves may not always be representative of the specific grossing protocols or specimen types used for molecular testing in clinical practice (eg, biopsies versus resection specimens). Evaluation on histological images from diverse clinical cohorts and including biopsy specimens remains a useful next step for validation of the LungCNN-TMB model described here. Molecular tumor heterogeneity represents another potential limitation, as there may theoretically be differences in the molecular profile of the specific tissue used for molecular testing relative to the specific portion of the tumor represented in the FFPE WSI. However, WSI-based molecular predictions may themselves be a tool that facilitates evaluation of tumor heterogeneity, by providing feature maps of histologic subtypes as well as highlighting features associated with specific molecular findings in one portion of tumor versus another. Although not available for this study, the use of immunotherapy response data to evaluate TMB classification accuracy would provide informative clinical validation. Lastly, in this study we predominantly explored the use of LungCNN-Histo output to predict TMB status based on the quantity of the histologic patterns. Future work to incorporate information from the complex patterns and spatial relationship within individual tumors remains an intriguing direction.

In this work we developed interpretable and novel approaches for TMB prediction in lung cancer, showing that such approaches are feasible and comparable in performance to weakly supervised models. We also developed and rigorously evaluated a model for histologic subtypes and features, capable of providing a feature map across WSIs. We hope this can provide a foundation for future work to build upon, including prediction of additional biomarkers and exploration of spatial relationships of histologic features within individual tumors. Lastly, we developed and compared hybrid approaches to evaluate the value of combining information across clinical data, weakly supervised feature learning, and comprehensive histologic pattern quantitation. While use of molecular prediction models in clinical practice will require further performance improvements and appropriate clinical validation, this work offers valuable demonstration of the potential for deep learning to provide efficient biomarker classification along with biological insights regarding histologic and molecular associations.

## Supplementary Information

Supplementary Information.
