# Supplementary material for: Comparative analysis of machine learning approaches to classify tumor mutation burden in lung adenocarcinoma using histopathology images
Source: Sci Rep. 2021 Aug 16;11:16605. doi: 10.1038/s41598-021-95747-4 (PMC8368039; doi:10.1038/s41598-021-95747-4)
Supplement: Supplementary file 1 — Supplementary Information. [file 41598_2021_95747_MOESM1_ESM.docx]

# Supplemental Figures and Tables

**Comparative analysis of machine learning approaches to classify tumor mutation burden in lung adenocarcinoma using histopathology images**

Apaar Sadhwani^†^, Huang-Wei Chang^†^, Ali Behrooz, Trissia Brown, Isabelle Auvigne-Flament, Hardik Patel, Robert Findlater, Vanessa Velez, Fraser Tan, Kamilla Tekiela, Ellery Wulczyn, Eunhee S. Yi, Craig H. Mermel, Debra Hanks, Po-Hsuan Cameron Chen, Kimary Kulig, Cory Batenchuk^‡^, David F. Steiner^‡^, Peter Cimermancic^‡^

^†^These authors contributed equally

^‡^These authors jointly supervised this work

# Contents:

# Supplemental Figure S1: STARD diagram with exclusion/inclusion criteria for the test sets.

**Supplemental Figure S2**: LungCNN-Histo performance confusion matrix across prediction classes.

**Supplemental Figure S3:** Percent consensus across pathologists for the test set annotations.

**Supplemental Figure S4:** Model performance comparison using a TMB threshold of 200 and 323.

**Supplemental Figure S5:** Feature correlation and importance.

**Supplemental Figure S6:** ​​Performance of the weakly supervised model (WS-S1) with varying amounts of training data.

**Supplemental Table S1:** Counts of patches by histologic feature used for training and testing the tumor histology model.

**Supplemental Table S2:** Hyperparameter description for LungCNN-Histo.

**Supplemental Table S3**: Hyperparameter description for the logistic regression model using histologic and clinical features to predict TMB status (LungCNN-TMB)

**Supplemental Table S4**: Hyperparameter description for the weakly supervised TMB model (WS-S1)

# Supplemental Figures


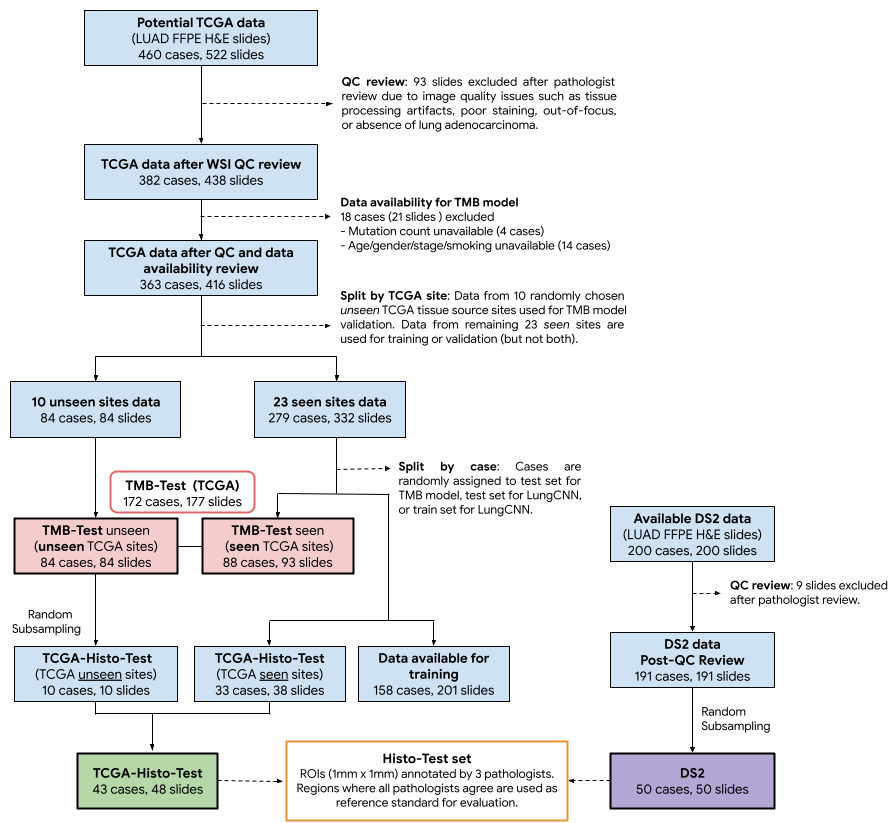


**Supplemental Figure S1:** STARD diagram with exclusion/inclusion criteria for the test sets. The histologic subtype model (LungCNN-Histo) was evaluated on TCGA slides as well as the external data source, DS2. All TMB models (histologic subtype-based, weakly supervised, and hybrid models) were trained and validated using TCGA data only (noting that mutation counts required for TMB model development or evaluation were not available for DS2). The TCGA data represents 33 tissue source sites that capture variations across sites such as tissue preparation and staining. We designated 10 tissue source sites as unseen and their data were used exclusively for final evaluation of models, thus representing a subset of TMB-Test. The data from the remaining 23 seen sites was split into a train set and test set by case.


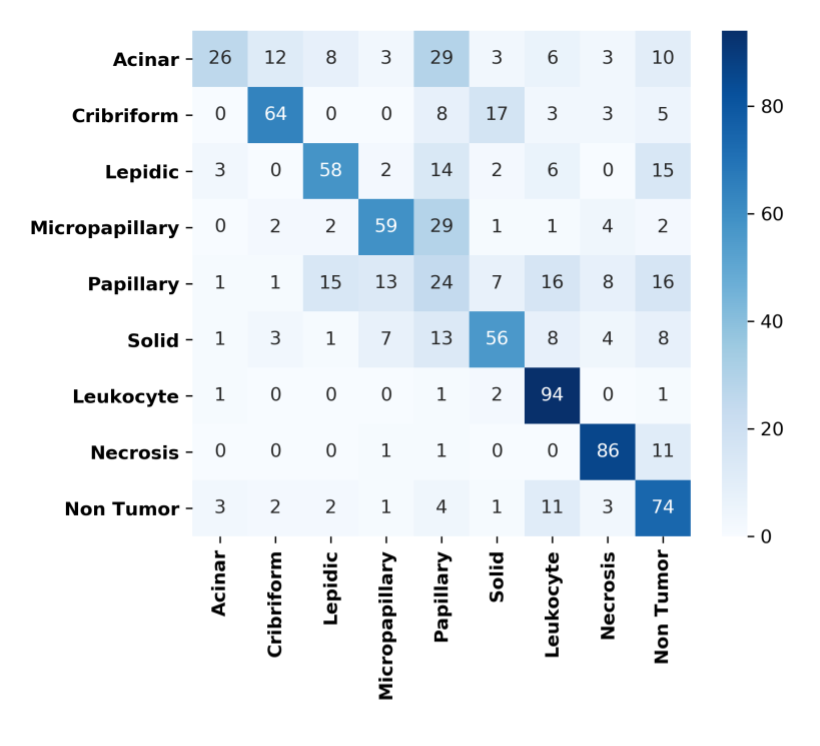


**Supplemental Figure S2**: LungCNN-Histo performance confusion matrix across prediction classes. Row labels represent consensus pattern labels based on annotations of the test set. Columns represent LungCNN-Histo predictions. Values represent the percentage of total patches for each row with the prediction specified by the columns. Patches correspond to consensus labeled patches of the complete test set (TCGA-Test plus DS2). Color scale corresponds to the numerical values as indicated.

**
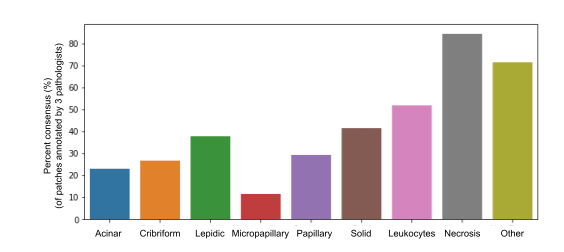
**

**Supplemental Figure S3:** Percent consensus across pathologists for the test set annotations. Data represents all patches within Histo-Test for which labels were provided by 3 pathologists. The denominator for each class is the number of patches labeled by at least one pathologist as the corresponding class.


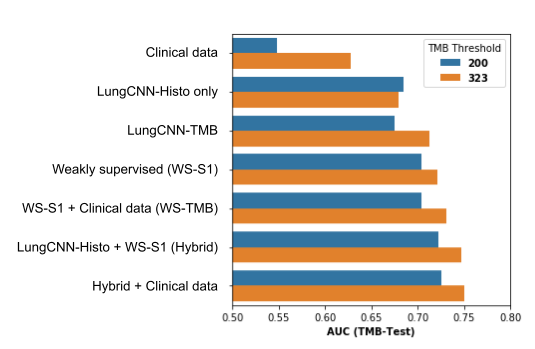


**Supplemental Figure S4:** Model performance comparison using a TMB threshold of 200 and 323. AUCs are plotted for TMB classification across all models developed. Clinical data plus LungCNN-Histo represents LungCNN-TMB. AUC: Area under the receiver operator characteristic curve


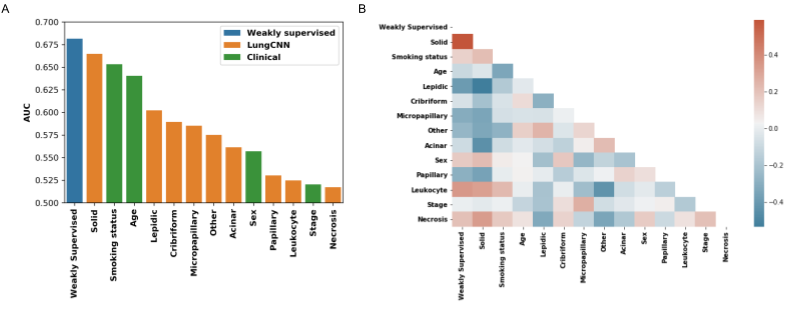


**Supplemental Figure S5:** Feature correlation and importance. (A) Correlation between features, ordered left to right by decreasing predictive value for TMB. (B) AUCs obtained by predicting TMB scores with the individual features indicated on the x-axis (using 10-fold cross-validation on the TMB-train set).


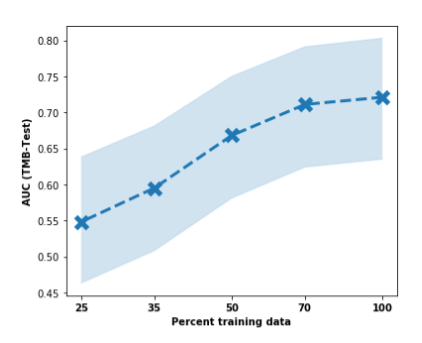


**Supplemental Figure S6:** ​​Performance of the weakly supervised model (WS-S1) with varying amounts of training data. The x-axis shows on a log scale the volume of data (as a fraction of the full training set for WS-S1 described in Table 1) used to train the model. The model fails to learn useful features when trained with less than 25 percent of the overall data. Thereafter, the performance improves with increasing data while its marginal gain decreases. Each “x” represents the average of 5 experiments and the shaded area represents 95% CI.

# Supplemental Tables

## **Supplemental Table S1:** Counts of patches by histologic feature used for training and testing the tumor histology model.

|  | **Train** | **Test** | |
| --- | --- | --- | --- |
|  | **TCGA LUAD** | **TCGA LUAD** | **DS2** |
| **Acinar** | 136316 | 133729 | 149908 |
| **Cribriform** | 319419 | 47517 | 59502 |
| **Lepidic** | 153623 | 33937 | 12985 |
| **Micropapillary** | 112816 | 18162 | 81988 |
| **Papillary** | 127963 | 48147 | 2524 |
| **Solid** | 616231 | 206765 | 215071 |
| **Leukocyte** | 111672 | 124550 | 69363 |
| **Necrosis** | 204187 | 140958 | 272036 |
| **Non Tumor** | 2832484 | 796825 | 701146 |
| **Total** | 4614711 | 1550590 | 1564523 |

## **Supplemental Table S2:** Hyperparameter description for LungCNN-Histo.

| **Hyperparameter** | **Description** | **Values** | **Optimal configuration** |
| --- | --- | --- | --- |
| Patch size | Height and width of each image patch | 256, 384, 512, 768, 1024 | 512 |
| Magnification | Image magnification at which the patches are extracted | 20X, 10X, 5X | 10X |
| Neural network architecture | Convolutional neural network architecture | InceptionV3, MobileNet, ResNet-50, ResNet-101 | InceptionV3 |
| Depth Multiplier | Multiplier on the depth of each convolution layer for downscaling the number of parameters in the default InceptionV3 architecture | 0.05, 0.08, 0.1, 0.2 | 0.1 |
| Batch size | Number of examples in each training batch | 8, 16, 32, 64 | 8 |
| Class sampling ratio | Relative ratio of classes at train time | Uniform, 2x/3x/4x weight on tumor classes | Uniform |
| Optimizer | The optimization algorithm used for model training | RMSProp | RMSProp |
| L2 regularization weight | Weight of the L2 loss used for regularization | 0.004, 0.0004, 0.00004 | 0.0004 |
| Initial learning rate | Initial learning rate used for the RMSPROP optimizer; decay rate was 0.99 every 20,000 steps | 0.001, 0.0001, 0.00001 | 0.0001 |
| Learning rate decay steps | Number of steps after which the learning rate is decreased by multiplying by the decay rate | 5000, 10000, 15000, 20000, 25000, 30000 | 25000 |
| Learning rate decay rate | The rate at which the learning rate is decayed after a fixed number of steps | 0.90, 0.95, 0.99 | 0.90 |
| Training steps | The number of steps for which the model is trained | 1000000 | 1000000 |

##

##

## **Supplemental Table S3**: Hyperparameter description for the logistic regression model using histologic and clinical features to predict TMB status (LungCNN-TMB)

| **Hyperparameter** | **Description** | **Values** | **Optimal configuration** |
| --- | --- | --- | --- |
| Balanced class weights | Whether to balance the weights corresponding to the two classes | True, False | False |
| Solver | The solver to use for fitting the logistic regression model | liblinear, lbfgs, saga | liblinear |
| Regularization penalty weight-inverse | The weight of log cross-entropy loss relative to regularization penalty | 0.1, 0.2, 0.4, 0.8, 1, 2, 4, 8 | 2 |
| Regularization penalty type | The type of regularization penalty to apply to model coefficients | L1, L2, ElasticNet | L2 |
| L1 ratio | Ratio of L1 to L2 penalty; applies only to ElasticNet | 0, 0.2, 0.4, 0.6, 0.8, 1 | 1 |

#

##

## **Supplemental Table S4**: Hyperparameter description for the weakly supervised TMB model (WS-S1)

| **Hyperparameter** | **Description** | **Values** | **Optimal configuration** |
| --- | --- | --- | --- |
| Patch size | Height and width of each image patch | 256, 384, 512, 768 | 512 |
| Magnification | Image magnification at which the patches are extracted | 20X, 10X, 5X | 10X |
| Maximum sequence length | Length of the sequence of patches used for training | 1, 4, 8, 16 | 8 |
| Neural network architecture | Convolutional neural network architecture | InceptionV3, MobileNet | MobileNet |
| Base depth | Number of channels in the first MobileNet layer | 16, 32, 64 | 32 |
| Maximum depth | Maximum number of channels in any MobileNet layer | 256, 512 | 256 |
| Batch size | Number of examples in each training batch | 8, 16, 32 | 16 |
| Loss | Loss function used for training | Softmax cross-entropy | Softmax cross-entropy |
| Optimizer | The optimization algorithm used for model training | RMSProp | RMSProp |
| L2 regularization weight | Weight of the L2 loss used for regularization | 0.004, 0.0004, 0.00004 | 0.0004 |
| Initial learning rate | Initial learning rate used for the RMSPROP optimizer; decay rate was 0.99 every 20,000 steps | 0.001, 0.0001, 0.00001 | 0.0001 |
| Learning rate decay steps | Number of steps after which the learning rate is decreased by multiplying by the decay rate | 5000, 10000, 20000 | 5000 |
| Learning rate decay rate | The rate at which the learning rate is decayed after a fixed number of steps | 0.90, 0.95, 0.99 | 0.90 |
| Training steps | The number of steps for which the model is trained | 500000, 1000000 | 500000 |
| Dropout | Whether to use dropout for convolutional layers (keep probability 0.8). Dropout always applied to the prelogits. | True, False | True |

# 
